# Myeloid Derived Hypoxia Inducible Factor 1-alpha Is Required for Protection against Pulmonary *Aspergillus fumigatus* Infection

**DOI:** 10.1371/journal.ppat.1004378

**Published:** 2014-09-25

**Authors:** Kelly M. Shepardson, Anupam Jhingran, Alayna Caffrey, Joshua J. Obar, Benjamin T. Suratt, Brent L. Berwin, Tobias M. Hohl, Robert A. Cramer

**Affiliations:** 1 Department of Microbiology and Immunology, Geisel School of Medicine at Dartmouth, Hanover, New Hampshire, United States of America; 2 Infectious Disease Service, Department of Medicine, Memorial Sloan-Kettering Cancer Center, New York, New York, United States of America; 3 Department of Microbiology and Immunology, Montana State University, Bozeman, Montana, United States of America; 4 Department of Medicine, University of Vermont College of Medicine, Burlington, Vermont, United States of America; University of Pittsburgh, United States of America

## Abstract

Hypoxia inducible factor 1α (HIF1α) is the mammalian transcriptional factor that controls metabolism, survival, and innate immunity in response to inflammation and low oxygen. Previous work established that generation of hypoxic microenvironments occurs within the lung during infection with the human fungal pathogen *Aspergillus fumigatus*. Here we demonstrate that *A. fumigatus* stabilizes HIF1α protein early after pulmonary challenge that is inhibited by treatment of mice with the steroid triamcinolone. Utilizing myeloid deficient HIF1α mice, we observed that HIF1α is required for survival and fungal clearance early following pulmonary challenge with *A. fumigatus*. Unlike previously reported research with bacterial pathogens, HIF1α deficient neutrophils and macrophages were surprisingly not defective in fungal conidial killing. The increase in susceptibility of the myeloid deficient HIF1α mice to *A. fumigatus* was in part due to decreased early production of the chemokine CXCL1 (KC) and increased neutrophil apoptosis at the site of infection, resulting in decreased neutrophil numbers in the lung. Addition of recombinant CXCL1 restored neutrophil survival and numbers, murine survival, and fungal clearance. These results suggest that there are unique HIF1α mediated mechanisms employed by the host for protection and defense against fungal pathogen growth and invasion in the lung. Additionally, this work supports the strategy of exploring HIF1α as a therapeutic target in specific immunosuppressed populations with fungal infections.

## Author Summary

Due to the limited treatment options and severity of invasive fungal infections, a better understanding of fungal-host interactions is needed for the development of new therapies. Recent studies have implicated a role for hypoxia inducible factor 1-alpha (HIF1α) in the regulation of inflammation and host defense responses to microbial pathogens. In this study, we discover that HIF1α is required for protection and murine survival to *Aspergillus fumigatus* pulmonary challenge. First, we observed that nuclear HIF1α protein levels are reduced in the murine corticosteroid immunosuppressed model of invasive pulmonary aspergillosis, suggesting its involvement in disease outcome. We then tested the hypothesis that HIF1α is required by innate immune effector cells to control/prevent *A. fumigatus* growth and invasion. Surprisingly, we observed that the role of myeloid HIF1α is not to mediate innate effector cell *A. fumigatus* killing directly, but rather to induce and maintain a protective immune response that ensures proper effector cell recruitment and survival at the site of infection. These findings provide a better understanding of host mechanisms involved in thwarting fungal pathogenesis, have implications for host susceptibility, and reveal the potential for novel treatment strategies involving HIF1α mediated signaling in the lung in immune suppressed patients.

## Introduction

Invasive fungal infections continue to take a toll on human health with high mortality and morbidity rates and increasing frequency [1]. The filamentous fungus *Aspergillus fumigatus* remains the most common cause of airborne invasive fungal infections and is the primary causal agent of invasive pulmonary aspergillosis (IPA) [2]. The persistence of sub-optimal IPA clinical outcomes is in part due to a less than optimal understanding of the *Aspergillus*-host interaction and toxicity associated with current antifungal drugs [3], [4], [5]. While much effort for treatment improvement is focused on identification of new antifungal drug targets and compounds, a complementary and important area of investigation is discovering therapies that improve host defense in immunocompromised patients [6].

Host defense to *A. fumigatus* challenge requires a functioning innate immune response with a strong dependence on neutrophils and other innate immune system effector cells, as their deficiency or defective function is a principal clinical risk factor for IPA [7], [8]. The timing and magnitude of neutrophil recruitment is pivotal for fungal clearance as a small delay in arrival leads to disease susceptibility [9], [10]. The recruitment of neutrophils to the site of infection requires a calculated interplay between multiple signaling chemoattractants and receptors that remain to be fully defined in the context of IPA. Alveolar macrophages, likely the first leukocyte exposed to conidia within the lung, induce the expression of pro-inflammatory cytokines, such as TNF, IL-1α/β, IL-6, GM-CSF, CXCL2, and CXCL1 in response to engagement of fungal PAMPs by macrophage PRRs such as toll-like receptors (TLRs) and dectin-1 [11], [12], [13]. Whether through MyD88-dependent or –independent signaling, the expression of these cytokines is mediated through the transcription factors NFκB, NFAT, and IRF's and are indispensible for proper clearance and preventing invasive disease [12]. In particular, the CXC chemokines defined by the amino acid sequence Glu-Leu-Arg (ELR) preceding the CXC motif, macrophage inflammatory protein 2 (MIP-2, murine CXCL2) and keratinocyte-derived chemokine (KC, murine CXCL1) are pivotal factors for neutrophil migration [14]. Neutrophils sense and respond to these inflammatory signals through surface expressed G-protein coupled receptors, cytokine receptors, adhesions (selectins and integrins), Fc-receptors, and innate receptors (TLRs and C-type lectins) [15]. Classical chemoattractant receptors expressed on the surface of neutrophils, including leukotriene B_4_, platelet-activating factor, and CXCR1 and CXCR2 are required for neutrophil recruitment and migration; mice deficient in these receptors are more susceptible to IPA [10], [16], [17], [18]. Once recruited to the lung and upon contact with hyphae, neutrophils induce degranulation, the respiratory burst, proteases, and antimicrobial peptides leading to both pathogen and host damage [9], [19], [20], [21]. The precise molecular mechanisms involved in neutrophil lung recruitment in response to *A. fumigatus* infection remain to be fully defined.

Pathogen driven inflammation and necrosis of tissue leads to development of microenvironments deficient in oxygen and nutrients, but there is a lack of knowledge regarding how host and pathogen responses to these deficiencies affect the outcome of pathogenesis [21]. Studies have implicated a role for hypoxia inducible factor 1 (HIF1α), a major regulator of the mammalian response to hypoxia, in the regulation of inflammation and host defense responses to microbial pathogens [22], [23], [24], [25]. However, the role of HIF1α in immune responses to lung microbial pathogens, and particularly fungi, is largely unknown. HIF1α is a heterodimeric protein whose α-subunit is stabilized under hypoxic conditions and translocated to the nucleus where it dimerizes with the β-subunit, referred to as the aryl hydrocarbon receptor nuclear translocator (ARNT, HIF1β) protein [26]. Once stabilized and in the nucleus, HIF1α binds to hypoxia response elements (HREs) of target hypoxia response genes including those involved in the processes of glucose metabolism, hypoxia, apoptosis, angiogenesis, and erythropoiesis [27]. HIF1α is regulated by prolyl hydroxylase's (PHD) through hydroxylation of its oxygen-dependent degradation domain and directs HIF1α for ubiquitin-dependent degradation by the von Hippel-Lindau (vHL) protein when oxygen is present [26], [28]. Activation of HIF1α requires basal levels of NFκB and in turn, NFκB activation is controlled by hypoxic inactivation of PHDs [29], [30]. Moreover, the activation of NF-κB in normoxia leads to up-regulation of HIF1α mRNA levels contrary to hypoxia increased protein levels [30]. This interdependence between HIF1α and NFκB supports a strong link between innate immunity and metabolism in controlling disease.

HIF1α plays an important role in innate immunity and host defense as myeloid cells have HIF-dependency for adaptation to hypoxic and inflamed microenvironments that develop during infection. For example, HIF1α is critical for regulating bactericidal activity of phagocytes against Group B *Streptococcus* [31]. In other pathogens, such as Group A *Streptococcus* and *Staphylococcus aureus*, presence and induction of HIF1α in myeloid cells, specifically macrophages and neutrophils, increases phagocytic activity and controls systemic spread of these pathogens [23], [25]. HIF1α has been suggested to be involved in the suppression of the angiogenic response by *A. fumigatus* in murine models of IA [32]. More recently, pulmonary HIF1α mRNA levels were observed to increase in response to the human fungal pathogen *Coccidioides immitis* [33].

Although the roles of HIF1α in myeloid cell bactericidal activity and inflammatory diseases are established, the function of HIF1α in the course of lung infections and particularly with fungi remains unclear. Therefore, we investigated the role of HIF1α following pulmonary challenge with *A. fumigatus* using a myeloid-specific lysozyme-M cre-recombinase driven HIF1α null mouse (HIF^C^) [22]. We observed that *A. fumigatus* challenge strongly induces HIF1α stabilization in wild-type immune competent murine lungs and macrophages; a process that is inhibited by administration of corticosteroids. Loss of myeloid HIF1α in otherwise immune competent mice resulted in a dramatic decrease in murine survival when challenged with *A. fumigatus* conidia. Surprisingly, rather than a role for HIF1α in mediating fungal killing by innate effector cells as previously observed with bacterial pathogens, the reduction in murine survival was in part mediated by a reduced number of neutrophils and innate immune effector cells early following fungal challenge. Reductions in these innate effector cells in the airways and lung in the absence of HIF1α were partially due to defective induction of the chemotactic signal CXCL1. These results support a role for HIF1α in initiating the correct inflammatory signal and immune response in order to prevent pulmonary fungal growth. Therefore, modulation of HIF1α signaling in specific immunocompromised patient populations is a potential area for therapeutic development.

## Results

### Corticosteroid treatment reduces HIF mRNA abundance and protein nuclear localization induced by *A. fumigatus* pulmonary challenge

**Figure 1:**
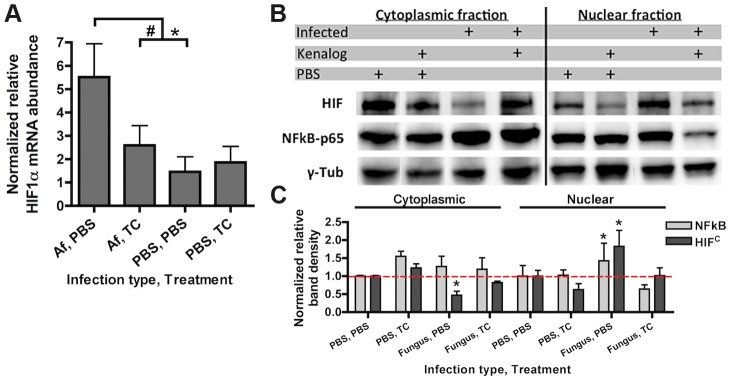
Steroid treatment reduces HIF mRNA abundance and protein nuclear localization induced by *A. fumigatus* infection. CD-1 mice were treated with triamcinolone (TC) or PBS (Treatment) 1 day prior to fungal inoculation. Mice were inoculated with either 7×10^7^ conidia or PBS i.t. (infection type) and lungs were collected for analysis at 24 hrs. A) mRNA abundance of *HIF1α* from the whole lung was analyzed by quantitative-RT-PCR. Combined 4 biological and 3 technical replicates for each transcript, using *hprt* as the housekeeping gene for normalization. Fold change is relative to one PBS, PBS biological replicate. B) Protein abundance of HIF1α and NFκB p65 subunit in the cytoplasmic and nuclear extracts from the whole lung were analyzed by Western Blot. Representative blot of two biological experiments shown. C) Quantitation of the band densities for HIF and p65 following normalization to γ-tubulin (γ-tub). Representative of two biological replicates with fold change relative to the PBS, PBS sample. * indicates a *P* value of <0.03 and # indicates a *P* value of 0.05 (unpaired two-tailed Students *t* test).

To determine whether HIF1α stabilization is part of the pulmonary innate defense response to pathogenic fungi, levels of HIF1α mRNA abundance and protein were analyzed in an immune competent murine model of fungal bronchopneumonia initiated by *A. fumigatus* challenge. Consistent with a potential role for HIF1α in defense against pulmonary fungal disease, *A. fumigatus* induced a three-fold increase in HIF1α mRNA abundance in the lung compared to PBS inoculated controls (Figure 1A). Accordingly, stabilization of HIF1α protein and increased nuclear localization occurred in murine lungs exposed to *A. fumigatus* conidia with greater HIF1α protein levels occurring in the cytoplasmic fraction of the PBS inoculated mice (Figure 1B). These results demonstrate two distinct effects of *A. fumigatus* challenge on HIF1α in the murine lung, the increase of HIF1α mRNA and an increase in nuclear HIF1α protein localization. In addition, HIF1α stabilization occurs *in vitro* with cultured macrophages exposed to *A. fumigatus* conidia and germlings in standard tissue culture conditions (Figure S1A). Taken together, in a healthy immune competent murine lung, HIF1α is stabilized in response to *A. fumigatus* pulmonary challenge, suggesting an important role for this protein in resistance to pulmonary fungal growth and subsequent infection.

Consequently, we next sought to determine whether activation of HIF1α was inhibited under conditions known to enhance susceptibility to *A. fumigatus* pulmonary growth and infection. We determined the effects of corticosteroids on HIF1α stabilization in response to *A. fumigatus* [34]. Although the effect of steroids on host immune cells has been focused on the role of NFκB, recently, the glucocorticoid dexamethasone was found to abrogate the activation of HIF1α in response to inflammation induced hypoxia [35], [36]. Intriguingly, corticosteroid treatment of mice significantly reduced mRNA induction of HIF1α two-fold when exposed to conidia of *A. fumigatus* (Figure 1A). Corticosteroid treatment also resulted in decreased levels of HIF1α protein with overall reduced levels in nuclear extracts in both *A. fumigatus* inoculated and uninoculated mice compared to immune competent mice (Figure 1B–C). Reductions in nuclear levels of the p65 subunit of NFκB in the corticosteroid treated mice were also observed, which has been reported previously, validating the chosen murine model (Figure 1B–C) [37], [38]. These results suggest that reductions in HIF1α nuclear levels may contribute to susceptibility of corticosteroid treated mice to *A. fumigatus*.

### Myeloid HIF1α is required for survival and clearance following *A. fumigatus* pulmonary challenge

**Figure 2:**
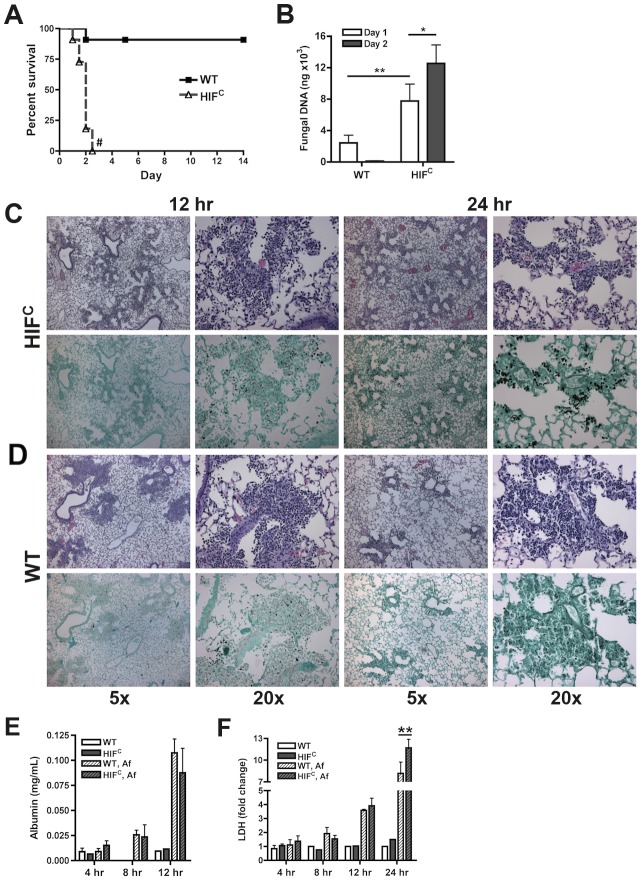
Myeloid HIF1α is required for fungal clearance and survival following *A. fumigatus* pulmonary challenge. Immune competent littermate (Cre-/HIF1α floxed: WT) and HIF^C^ mice received 7×10^7^ conidia i.t. and were A) monitored for survival (Log-Rank Test, ^#^ = p<0.0001, repeated twice) and B) Fungal burden on days 1 and 2 post challenge. Data represent 5 biological and 3 technical replicates for each time point. Representative histology of H&E or GMS stained lung sections from C) HIF^C^ and D) WT mice at 12 and 24 hr post challenge. Left image (5×), right image (20×). Levels of E) Albumin and F) LDH were measured in the BALFs at 4, 8, 12, and 24 (LDH) hrs of WT and HIF^C^ mice challenged with 7×10^7^ conidia (Af) or PBS (4–8 biological replicates for each time point). * indicates *P* value of <0.02, ** indicates *P* value of <0.05 (unpaired Students *t* test).

To delineate the function of HIF1α in pulmonary host defense to *A. fumigatus*, we utilized a conditional knockout system to strongly reduce HIF1α levels in the myeloid compartment [22]. The conditional mice (HIF^C^) expressed Cre recombinase under the control of the lysozyme M promoter in combination with the loxP flanked exon2 of the HIF1α gene. HIF1α levels were significantly reduced in myeloid derived cells, especially macrophages and neutrophils (Figure S1B) [22]. Immune competent mice deficient in myeloid HIF1α (HIF^C^) were strikingly more susceptible to *A. fumigatus* pulmonary challenge compared to littermate controls, with 100% mortality occurring in HIF^C^ mice by day 3 of the experiment and statistically different mortality by day 2 post- fungal inoculation (p = 0.0007) (Figure 2A, p<0.0001). HIF^C^ mice had increased fungal burden at 24 and 48 hrs post inoculation compared to littermate controls that began clearing the conidial inoculum after 24 hrs as measured by quantitative real-time PCR analysis of the fungal 18S rDNA gene (Figure 2B) [39]. Dissemination to the liver and kidney was also increased in HIF^C^ mice (data not shown).

Histopathology of the lungs 8 hrs post-fungal inoculation revealed no apparent difference in the number of conidia in the lungs of HIF^C^ and littermate control mice (Figure S2A). However, decreased levels of cellular infiltrate and inflammation were apparent in HIF^C^ mice *A. fumigatus* challenged mice (Figure S2A), and this phenotype was also observed at 12 hrs post inoculation (Figure 2C–D). However, the difference in the cellular infiltrate and inflammation was dramatically greater in the littermate controls at 12 hrs post inoculation consistent with a self-resolving fungal bronchopneumonia in these immune competent mice. At later time points post fungal inoculation, 24 and 48 hrs, littermate control mice were able to clear and contain the infection with mostly fungal debris remaining in the lung at 48 hrs (Figure 2C–D & Figure S2B), while the HIF^C^ mice develop invasive disease with uncontrolled hyphal growth. Additionally, the HIF^C^ mice develop increased levels of pulmonary damage, with significantly more lactate dehydrogenase (LDH) apparent in bronchoalveolar lavage fluid (BALF) at 24 hrs post challenge, but with no marked differences in vascular leakage determined by albumin BALF levels (Figure 2E–F). These results suggest that there is a defect in the innate immune response early during the response to *A. fumigatus* challenge in the HIF^C^ mice that renders them unable to clear and prevent fungal growth and host tissue damage.

### Susceptibility of HIF^C^ mice is not due to a defect in uptake or killing by pulmonary myeloid effector cells

**Figure 3:**
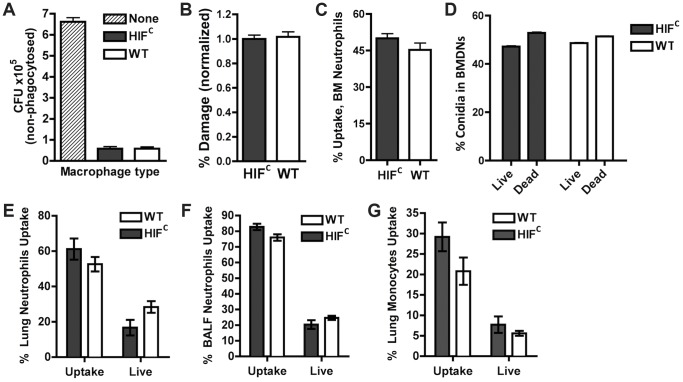
HIF1α is not required for phagocytic uptake or killing of *A. fumigatus* by macrophages, monocytes, and neutrophils. BMDMs (A,B) and BMDNs (C,D) from littermate (WT) and HIF^C^ mice were incubated with germlings (A,B) or FLARE conidia (C,D) in a 9∶1 ratio for 3 hrs and CFU (A), Damage by XTT (B), or Uptake (C) and Killing (D) by FACs analysis. Uptake and killing *in vivo* of FLARE conidia by neutrophils (lung,E:BALF,F) and monocytes (G) 12 hrs following challenge with 3×10^7^ FLARE conidia. Uptake bars represent both live and dead conidia engulfed and live bars represent only engulfed live conidia by the specific cells. Shown are cumulative data from three independent studies. The data are expressed as plus SEM. * indicates *P* value of <0.02 (unpaired two-tailed Students *t* test).

Previous reports on HIF1α's function during bacterial infection determined the importance of its activation and presence for neutrophil and macrophage-mediated pathogen killing [22], [23], [25]. We therefore sought to determine if the susceptibility of the HIF^C^ mice to *A. fumigatus* challenge was due to an overall defect in innate effector cell mediated fungal killing. First, utilizing *ex vivo* bone marrow derived macrophages (BMDMs) from HIF^C^ and littermate control mice, no difference in the ability of these cells to phagocytose conidia was observed between genotypes (Figure 3A). Additionally, there was no difference in the BMDM's ability to cause damage to phagocytosed conidia as measured by overall metabolic activity of the conidia phagocytosed by the BMDMs (Figure 3B).

To confirm these results *in vivo* in the context of appropriate inflammatory cues needed for full activation of innate effector cells, we generated a fluorescent *Aspergillus* reporter (FLARE) strain in the CBS144.89 (CEA10) wild-type background through ectopic insertion of TdTomato driven by the *A. nidulans gpdA* promoter. A single ectopic insertion of the construct by Southern blotting and FLARE based viability were confirmed as previously reported for the AF293 FLARE strain (Figure S3) [40]. *A. fumigatus* FLARE conidia contain two fluorophores that allow leukocytes to be distinguished based on their ability to engulf and/or kill conidia. The TdTomato fluorescence expressed by the conidia is lost upon conidia death, whereas the other fluorophore (AF633 or BV421) coating the conidia is stable even at low pH allowing for tracking of the conidia within leukocytes, whether dead or alive. Utilizing the FLARE strain, which allows real time measurement of conidial uptake and viability *ex vivo* and *in vivo*, we observed that HIF1α was not required in bone marrow derived neutrophil (BMDN) mediated uptake and killing of *A. fumigatus* conidia (Figure 3C–D) [40].

In agreement with *ex vivo* observations with single cell types derived from the bone marrow, inoculation of the FLARE strain into the immune competent murine model also revealed no significant difference in the ability of neutrophils or monocytes to engulf or kill conidia (Figure 3E–G). These surprising results demonstrate that HIF1α is not required for innate effector cell mediated killing following challenge with a fungal pathogen, which is in contrast to what has been determined with bacterial pathogens *ex vivo* and in skin models where HIF1α is critical for bactericidal activity of these effector cells [23], [25]. These results suggest unique HIF1α mediated mechanisms are employed by the host for protection and defense against fungal pathogen growth and invasion in the lung.

### HIF^C^ mice have decreased numbers of lung neutrophils early during infection

**Figure 4:**
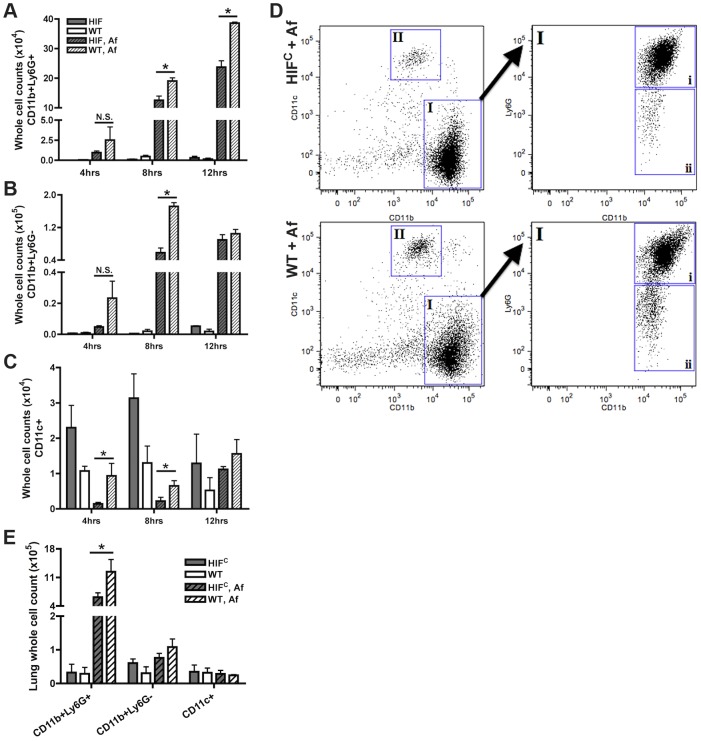
Mice deficient in myeloid HIF1α have significantly less neutrophils in BALF and lung tissue early during lung infection. FACs analysis of cell recruitment at 4, 8, and 12 hrs post challenge using CD11b, Ly6G, and CD11c fluorescent antibodies for staining of cells in the BALF (A, B, C, D) or lung (E, 8 hrs only) of littermate (WT) and HIF^C^ mice inoculated with 7×10^7^ conidia (Af) or PBS. Representative experiment of three (BALFs) or two (Lung digests) showing N = 4 for inoculated and N = 2 for mock. * indicates a *P* value of <0.03 (unpaired Students *t* test). Neutrophils are designated as CD11b+Ly6G+ (A and D, gate I, expanded gate I,i). Monocyte-like cells are designated as CD11b+Ly6G− (B and D, gate I, expanded gate I,ii). Macrophages are designated as CD11c+ (C and D, gate II). D) Representative FACs plots of WT and HIF^C^ infected mice (Af). Main plots are CD11b v. CD11c and expanded cell population gate I plot is CD11b v. Ly6G.

One factor determining the outcome of *A. fumigatus* lung infection is the timing and recruitment of neutrophils into the lung [8], [9], [41]. Due to the decrease in inflammation observed in the histology of the HIF^C^ *A. fumigatus* challenged mice, we quantitatively analyzed the level of innate effector cells through analysis of BALF and lung cellularity at early time points following fungal challenge. Consistent with the qualitative histopathology observations, the overall BALF and lung cellular infiltrates of inoculated littermate mice was greater than the HIF^C^ mice at all time points examined (Figure 4). At 4 and 8 hrs post fungal inoculation littermate mice displayed higher numbers of monocyte-like cells (CD11b+Ly6G−) and macrophages (CD11c+) in the BALF and at 8 hrs within the lung (Figure 4B–C,E). Interestingly, HIF^C^ mock treated mice tended to have modestly higher levels of macrophages than littermate controls, though not always statistically significant (Figure 4C,E). More importantly, the main innate effector cells known to be required for clearance and defense against *A. fumigatus*, neutrophils and inflammatory monocyte-like cells, were consistently ∼2–3 fold lower in HIF^C^ mice following fungal challenge at 4, 8 and 12 hrs in the BALF and at 8 hrs in the lung (Figure 4A,E p<0.02). These data suggest that HIF^C^ mice are defective in overall effector cell population numbers early following fungal challenge in the lung and that this may contribute to their inability to control fungal growth and tissue damage.

### HIF^C^ neutrophils have increased levels of apoptosis

Considering that early following fungal challenge there is precedence for the requirement of neutrophils and inflammatory monocytes in terms of infection outcome, we next sought to determine how HIF1α is involved in the neutrophil response following fungal challenge. During inflammation, the absence of HIF1α in effector cells is characterized by depletion of ATP stores due to a failure to switch from oxidative to glycolytic metabolism, which also results in an increase in toxic levels of ROS [42]. However, neutrophils rely strongly on glycolytic metabolism even though they have the capacity for aerobic respiration, supporting a major role for HIF1α in maintenance of normal neutrophil function [43]. Therefore, we sought to determine whether there was a defect in survival of HIF1α-deficient neutrophils in the presence and absence of fungal stimulation.

**Figure 5:**
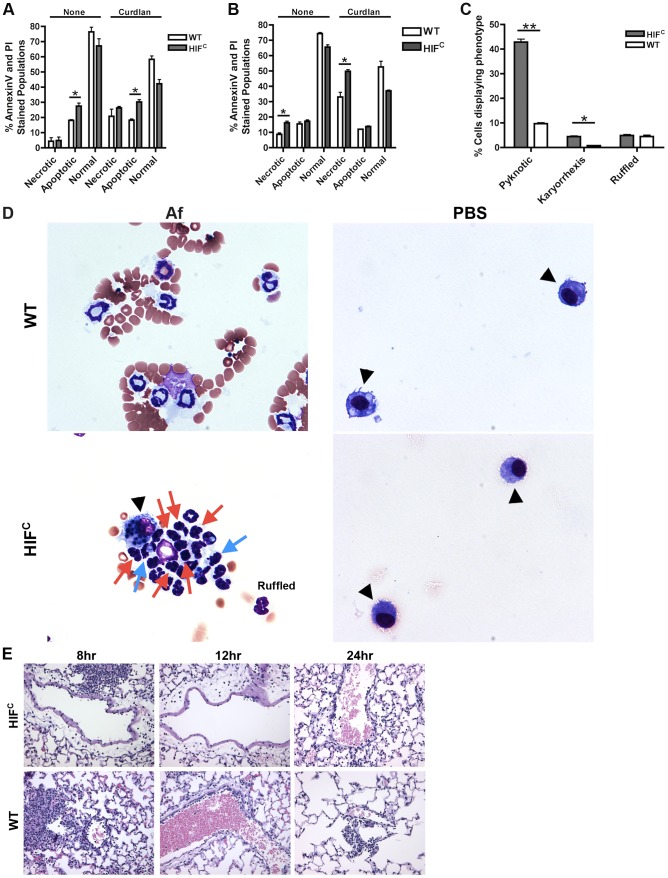
HIF^C^ neutrophils have increased levels of apoptosis and necrosis and decreased endothelial attachment. Littermate (WT) and HIF^C^ BMDNs were stimulated with PBS or 100 ug/mL curdlan in TC media for 5 hr (A) or 22 hr (B) and AnnexinV/PI staining was conducted using FACs (combined two biological replicates). Av+PI+ (necrotic), Av+PI− (apoptotic), Av−PI− (normal). C) Percent neutrophils demonstrating pyknotic or karyorrhexis nuclei and ruffled membranes was determined from cytospins from littermate (WT) and HIF^C^ mice at 8 hr post challenge with 7×10^7^ conidia (Af) or PBS. Data represent the mean from 2 independent biological replicates from 4 mice/genotype in each experiment. (D) Representative images of the cytospins depicting cell phenotypes. Red arrows indicate pyknotic neutrophils, blue arrows indicate neutrophil karyorrhexis, ruffled neutrophils labeled as ruffled, and black arrow heads designate macrophages. All images = 60×. (E) Histology vasculature images displaying margination of neutrophils at 20×. * indicates *P* value of <0.05 ** indicates *P* value of <0.01 (unpaired Students *t* test).

Agreeing with previously reported data [44], we determined that murine neutrophils deficient in HIF1α have increased cell death compared to wild-type control neutrophils with HIF1α deficient neutrophils exhibiting increased apoptosis at 5 hrs and increased necrosis at 22 hrs in the absence and presence of stimulation with the fungal β-glucan derivative curdlan (Figure 5A–B). Examination of the cellular infiltrates from BALFs of mice 8 hrs post *A. fumigatus* challenge revealed an increase in apoptotic neutrophils visualized by increased pyknotic nuclei and karyorrhexis of HIF^C^ compared to wild-type neutrophils (Figure 5C–D) [45], [46], [47]. There was no difference in the number of wild-type and HIF^C^ neutrophils with ruffled membranes, a marker of neutrophil activation (Figure 5C–D) [48]. These data correlate with the increase in LDH observed in the HIF^C^ mice (Figure 2F).

### The defect in pulmonary neutrophil numbers in the HIF^C^ mice correlates to reduced chemotactic and survival signals

Previous reports have identified a role for HIF1α in tissue adhesion, migration and invasion by neutrophils and macrophages at sites of infection and inflammation [22], [49]. In the HIF^C^ mice, we observed a decrease in the level of margination on the vessel walls compared to the littermate mice (Figure 5E). Margination is a prerequisite for neutrophil transendothelial migration (TEM) from the capillaries into pulmonary tissue [50]. There is much controversy as to the requirement for selectin-mediated rolling in order for TEM to occur, but chemokine production due to stimulation from foreign agents is known to increase margination and TEM [51], [52]. In addition to the lack of margination, the migration of the inflammatory cells away from the vessels is minimal in the HIF^C^ mice compared to littermate controls in which the cells have progressed into the alveoli (Fig. 2). These results demonstrate a defect in the HIF^C^ neutrophils ability to sense and migrate towards conidia within the lung environment.

**Figure 6:**
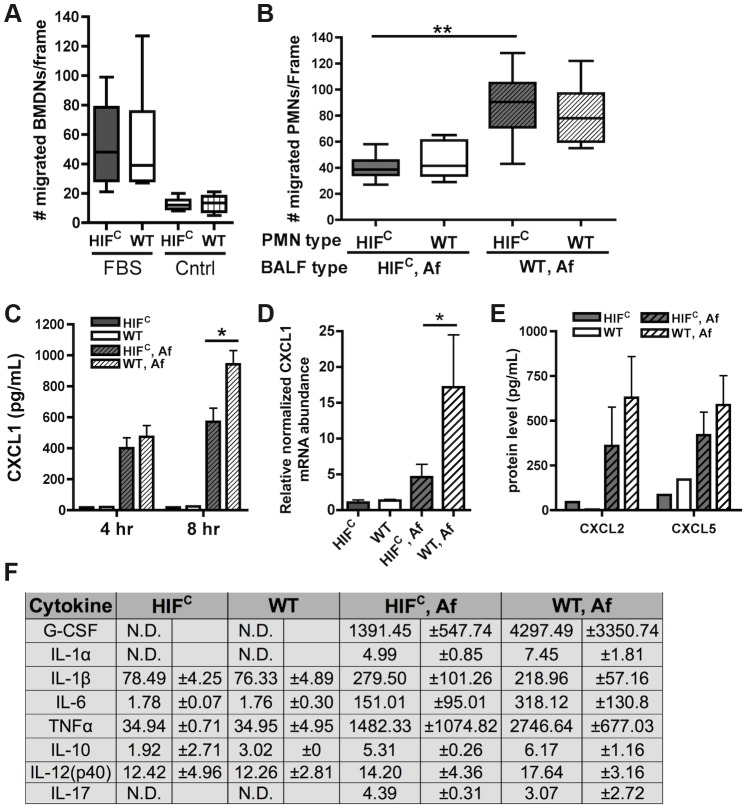
Decreased neutrophil levels in HIF^C^ mice correlates to decreased production of CXCL1 and not migration defects. Migration of littermate (WT) and HIF^C^ BMDNs in the presence of (A) RPMI media containing FBS or nothing or (B) 12 hr BALF from HIF^C^ or WT mice inoculated with 7×10^7^ conidia (Af) was measured using a 3 µm transwell chamber. (C) *In vivo* CXCL1 protein was measured in BALFs from 4 and 8 hr infections using an ELISA from AssayBiotech. (D) WT and HIF^C^ BMDMs were incubated with *A. fumigatus* conidia in a 10∶1 ratio for 8 hrs. mRNA abundance of *cxcl1* was determined using quantitative RT-PCR, normalized to *rpl13a*, and relative to the HIF^C^ sample (3 biological and 3 technical replicates). (E) *In vivo* CXCL2 and CXCL5 protein was measured in BALFs from 8 hr infections. (F) *In vivo* cytokine protein production was measured in BALFs from the same model in (B) using a cytokine luminex bead array from BioRad. (C,E) Data represent 3–4 biological replicates and depicted as mean plus SEM (N.D. = not detectable). * indicates a *P* value of <0.03 and ** indicates a *P* value of <0.05 (unpaired Students *t* test).

Therefore, we next sought to determine if there was a defect in neutrophil migration or chemotactic signaling in HIF^C^ mice that could account for decreased neutrophil numbers *in vivo* in response to *A. fumigatus* challenge. In response to the general chemotactic signal fetal bovine serum (FBS), HIF^C^ neutrophils were able to migrate to the same capacity as littermate neutrophils *ex vivo* (Figure 6A). Importantly, this assay was conducted within the time window during which there was no difference in apoptosis between WT and HIF^C^ neutrophils. Consequently, the migration results indicated that a defect in the production of chemotactic/cell survival signals at the infection site or the neutrophil response to tissue-specific signals may be the cause of reduced PMN numbers *in vivo*.

Since HIF^C^ neutrophils were competent to migrate in response to general chemotactic factors (Figure 6A), we next determined if there was a defect in the production of chemotactic signals during fungal pulmonary challenge that would result in decreased migration or cell survival. Utilizing the BALFs of *A. fumigatus* challenged HIF^C^ and littermate mice as the source of chemotactic factors in the transwell migration assay, we observed that littermate and HIF^C^ neutrophils were defective in migration towards the HIF^C^ BALF, but not towards littermate control BALF. This result indicated there was a missing chemotactic component in the HIF^C^ but not littermate control BALF (Figure 6B). These results support a defect in chemotactic or cell survival signal production in HIF^C^ mice following *A. fumigatus* pulmonary challenge.

Cytokine/chemokine analysis of the 12 hr BALFs from *A. fumigatus* challenged mice indicated decreased production of the pro-inflammatory cytokines G-CSF, IL-1α, IL-6, and TNF with no difference in production of the anti-inflammatory cytokine IL-10 (Figure 6F). There was no observed difference in the production of IL-17 and IL-12p40 between the WT and HIF^C^ challenged mice (Figure 6F). The overall response in the HIF^C^ mice at 12 hrs post challenge did not deviate from the usual Th1 protective response with Th2 specific cytokines either unchanged (IL-10) or undetectable (IL-4, data not shown). The production of one of the major neutrophil chemotactic cytokines CXCL1 was decreased in the HIF^C^ inoculated mice compared to littermate controls early following fungal challenge (Figure 6C). This reduction in CXCL1 correlated directly with the quantitative neutrophil defect found during early time points following fungal challenge (Figure 4A).

HIF1α is directly required for CXCL1 mRNA levels as the mRNA abundance in response to *A. fumigatus* is significantly decreased in macrophages deficient in HIF1α (Figure 6D). Whether the HIF1α regulation of CXCL1 mRNA levels is at the transcriptional or post-transcriptional levels remains unclear, however, analysis of the DNA sequence upstream of the CXCL1 start codon revealed multiple potential HRE elements (Figure S5A). Importantly, the production of CXCL2 [53] and CXCL5 [54], other neutrophil chemokines detected by CXCR2, was not different between the WT and HIF^C^ inoculated mice (Figure 6E). Additionally, no statistically significant difference in the mRNA abundance of the receptors CXCR2, TLR4, and Dectin-1 were observed between the WT and HIF^C^ neutrophils (Figure S5B). Taken together, these results support a direct role for HIF1α in production of cytokines early in the pulmonary response to *A. fumigatus* challenge. They also support the hypothesis that the neutrophil quantitative BALF defect in the HIF^C^ mice is due in part to decreased levels of CXCL1 and/or other pro-inflammatory cytokines.

### Neutrophil levels and survival of HIF^C^ mice can be restored by addition of physiologically relevant amounts of CXCL1 during early infection

**Figure 7:**
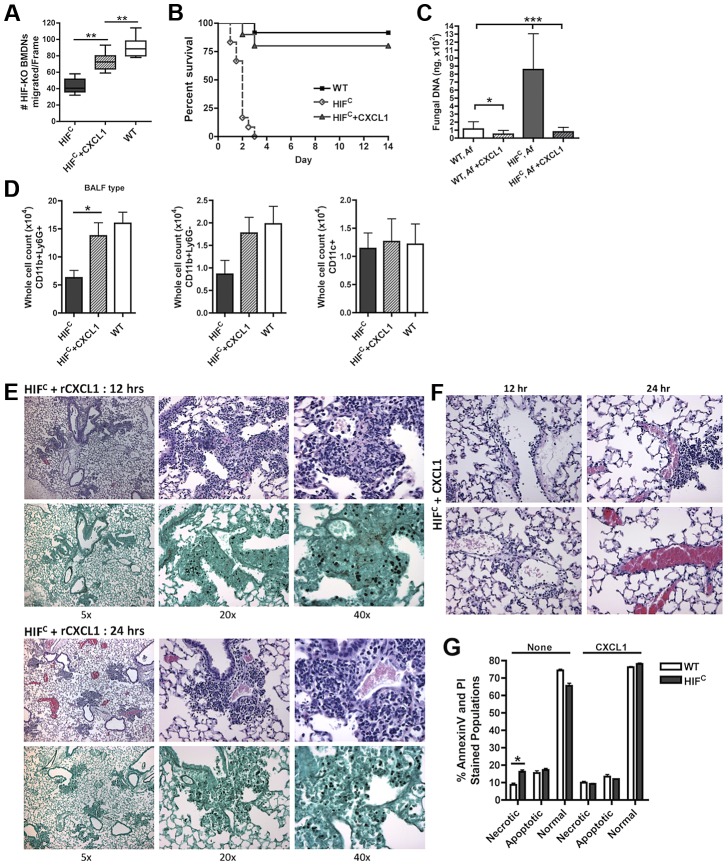
Restoration of physiologically relevant amounts of CXCL1 during infection restores neutrophil levels and survival of HIF^C^ mice. (A) BMDNs migration from littermate (WT) and HIF^C^ mice in the presence of 12 hr BALF from HIF, HIF+ 600 pg rCXCL1, and WT mice inoculated with 7×10^7^ conidia was measured using a 3 µm transwell chamber. Immune competent WT and HIF^C^ mice received 7×10^7^ conidia (Af) i.t. and were treated with 50 ng rCXCL1 4 hr post conidial challenge and monitored for (B) survival (p<0.002, 2 experiments: 12 mice total), (C) fungal burden at 36 hrs (4 biological, 3 technical reps), and (D) FACs analysis of BALF cellularity including: CD11b+Ly6G+ neutrophils, CD11b+Ly6G- monocyte-like, and CD11c+ macrophages, (N = 4 per group, representative of two experiments). Representative histology of H&E and GMS stained sections of the HIF^C^+CXCL1 mice (E) at 12 hr and 24 hr and H&E sections of (F) 12 and 24 hr vasculature (20×). (G) WT and HIF^C^ BMDNs were stimulated with PBS or 60 pg rCXCL1 for 22 hrs and AnnexinV/PI staining was conducted using FACs (combined two biological experiments). Av+PI+ (necrotic), Av+PI− (apoptotic), Av−PI− (normal).

We next sought to determine if the HIF^C^ phenotype was due, at least in part, to the marked reduction in CXCL1 levels. Mice deficient in CXCR2 (ligands CXCL1 (KC), CXCL2 (MIP2), CXCL5 (LIX)) develop invasive aspergillosis resulting from delayed neutrophil influx that allows conidial germination [10], [55]. Additionally, the transient expression of CXCL1 during invasive aspergillosis causes an earlier and increased number of neutrophils in the lung that results in improved host defense and outcome of *Aspergillus* infection [55]. In order to define the involvement of CXCL1 in neutrophil migration, LPS-free recombinant CXCL1 was added to the BALF of *A. fumigatus* challenged HIF^C^ mice to the level that was determined in the littermate BALF (Figure 6C). The addition of physiological levels of CXCL1 largely restored the neutrophil migration defect observed with *A. fumigatus* challenged HIF^C^ BALF (Figure 7A, p<0.001).

This significant restoration of neutrophil migration by CXCL1 led us to examine if the addition of recombinant CXCL1 to the HIF^C^ *A. fumigatus* challenged mice could restore neutrophil levels and murine survival. Previously, kinetics of neutrophil recruitment after 4 hrs by the intratracheal instillation of varying amounts of CXCL1 to the lung was demonstrated [56]. Doses of 30 ng and 100 ng of CXCL1 were demonstrated to induce ∼1.5 and ∼3.5 fold increases in neutrophil recruitment over PBS treatment, respectively [56]. Based on these previously published results, we utilized a dose of 50 ng recombinant CXCL1 for intratracheal instillation. The addition of CXCL1 to the HIF^C^ *A. fumigatus* challenged mice restored neutrophil numbers in the BALF back to levels of littermate inoculated mice and this restoration correlated with a significant increase in murine survival and improved outcome of infection based on the observed decrease in fungal burden and tissue damage (Figure 7B–C,E,F). The addition of CXCL1 also restored the inflammatory monocyte-like levels back to littermate controls, but not the CD11c+ macrophages, which was expected, as they are known to not respond to CXCL1 (Figure 7D, & Figure S4). These results demonstrate a unique requirement and mechanism for HIF1α control and induction of CXCL1 in response to *A. fumigatus* challenge in the lung.

Due to the multifunctional role of some cytokines in chemotactic and angiogenic responses and the observed increase in apoptosis/necrosis in the HIF^C^ neutrophils, we determined if the addition of recombinant CXCL1 affected survival of the HIF1α deficient neutrophils. Adding physiologically relevant levels of recombinant CXCL1 to the HIF^C^ neutrophils decreased their time-dependent level of apoptosis and necrosis *ex vivo* (Figure 7G). This demonstrates that CXCL1 signaling promotes and restores HIF1α neutrophil survival providing support for the requirement of HIF1α-dependent production of CXCL1.

## Discussion

In this study, we uncover a novel and essential function for myeloid HIF1α in protection against pulmonary *A. fumigatus* challenge. We present findings that myeloid HIF1α is required for initiating protective inflammatory signals and responses to control *A. fumigatus* growth and host tissue damage. These data strongly suggest a role for HIF1α in providing host protection to pulmonary fungal disease, provides a deeper understanding of the fungal-host interaction in the lung, identifies a new genetic factor critical for resistance to pulmonary murine fungal infections, and argues for further investigation into the therapeutic potential of HIF1α modulation for fungal disease.

Importantly, our results demonstrate a requirement for myeloid HIF1α in murine survival to *A. fumigatus* pulmonary challenge. The kinetics and final outcome of murine survival in the absence of myeloid HIF1α is striking. Mortality in HIF^C^ mice does not appear to be due to an increased inflammatory response as there is less overall inflammation in the lung at the time points analyzed in our model. Upon examination of the lung, there appears to be no gross defects in the vasculature in the HIF^C^ mice (also suggested by BALF albumin measurement), but as infection progresses and fungal burden increases an increase in the level of damage occurring in the lung in the absence of HIF1α occurs as evidenced by the increase in LDH levels. We cannot currently rule out systemic effects of HIF^C^ loss on murine survival, however, there was a trend toward increased fungal dissemination to the kidneys and liver in the HIF^C^ mice that could contribute to the rapid mortality (data not shown). The increased mortality therefore is likely due to the combination of the dysregulated host response and subsequent increased fungal burden following *A. fumigatus* challenge.

Perhaps most surprisingly, given previous studies in bacterial pathosystems, HIF1α was not required for direct killing of *A. fumigatus* conidia *ex vivo* or *in vivo* by innate effector cells. This is in contrast to the requirement for HIF1α in killing of multiple gram-negative and –positive bacteria in epidermal wound models of infection [22], [23], [25]. As HIF^C^ neutrophils are not defective in their ability to elicit the respiratory burst [23], it is perhaps not surprising that no difference in their ability to kill *A. fumigatus* conidia was observed due to the known conidial susceptibility to reactive oxygen species [57], [58]. Additionally, we cannot rule out that the ability to kill conidia may be due to a compensatory HIF2α mechanism, as it has distinct and overlapping biological roles with HIF1α [59]. Importantly, these observations demonstrate the plasticity of HIF1α regulation between different tissue microenvironments and with fungal and bacterial organisms. As the phenotypes of innate cells differ between tissues within the host [60], it will be important to determine if the findings presented here occur with other pathogens such as the bacteria examined in the epidermal model that are also potent lung pathogens.

Though HIF1α does not seem to regulate innate mediated killing of *A. fumigatus* conidia per se, our data suggest a required role for HIF1α in controlling *A. fumigatus* infection through modulation of the infection microenvironment that drives the timing, recruitment, and survival of neutrophils at early critical time points following *A. fumigatus* challenge. Recruitment of myeloid cells during epidermal inflammation is known to require HIF1α and is reported to be due to decreased HIF1-dependent integrin expression [22], [49]. This may partially account for the decreased margination observed in the HIF^C^ mice challenged with *A. fumigatus*, however, due to the increased percentage of neutrophils in the pulmonary capillary blood and the smaller size of the capillaries, the importance of integrin and selectin binding for pulmonary TEM to occur is debated [61], [62], [63]. Once at the site of inflammation, the ability of neutrophils to remain metabolically competent in low oxygen inflammatory environments is dependent upon the induction of the glycolytic pathway directly regulated by HIF1α [64]. In the absence of HIF1α and oxygen, neutrophils cannot maintain high ATP levels and as a consequence, undergo apoptosis [65]. The delay in the time-dependent recruitment of neutrophils and/or their apoptosis is partially responsible for the increased occurrence of conidial germination in the HIF^C^ mice [9]. The HIF^C^ *A. fumigatus* challenged mice also had decreased levels of CD11b+Ly6G− inflammatory monocyte-like cells, which have recently been implicated in inflammatory conditioning for neutrophil functions in the lung and conidial killing [41], and are also likely involved in the HIF^C^ phenotype.

The effects of myeloid HIF1α loss on effector cell recruitment and survival appear to be driven in part by defects in the production of pro-inflammatory cytokines. Though our data strongly support a major role for the neutrophil chemoattractant CXCL1 in mediating the HIF^C^ phenotype, other neutrophil chemoattractants/receptors that are known to be involved in neutrophil recruitment during various lung infections may be involved in the HIF1α phenotype including CCL3-CCL6-/CCR1 [66], C5a/C5aR [67], and LTB4/LTB4R1 [68], [69]. However, production of the other CXCR2 ligands CXCL2 [53] and CXCL5 [54] that are known to have roles in neutrophil recruitment were not different within the BALF of WT and HIF^C^ mice. Untangling the signal transduction cascades mediated by HIF1α, including how it is activated in response to *A. fumigatus* challenge, is an important future direction of this research.

Downstream of pathogen recognition, regulation of CXCL1 transcription through NFκB is a known mechanism for CXCL1 induction in response to inflammation [70]. Given the dual regulation of multiple genes by HIF1α and NFκB, it is perhaps not surprising that a role for HIF1α in CXCL1 regulation exists. Loss of CXCL1 mRNA levels in HIF1α macrophages and the presence of putative HREs in the CXCL1 promoter further support a direct role for HIF1α in control of CXCL1 murine lung levels in response to *A. fumigatus* challenge. Importantly, it appears that in the context of lung infection that HIF1α may play a more dominant role in myeloid activation of CXCL1 and the amplification of the response. This is based on previous demonstration that amplification of CXCL1 levels occurred only in epithelial cells that had constitutively active HIF1α over NFκB activation alone [71]. Given the decrease in IL-1 family members in the HIF^C^ mice, we hypothesize that IL-1 signaling may be play a critical function in these signal transduction cascades. Considering that transgenic expression of CXCL1 during the course of infection improved fungal burden and survival during infection with *A. fumigatus* [55], understanding the exact mechanism for HIF1α regulation of CXCL1 is of great importance.

Another important future direction is the cell compartment primarily producing CXCL1 and the effects of paracrine and autocrine signaling to regulate the observed effector cell phenotypes. Due to the ability of CXCL1 to reduce the apoptotic phenotype of the HIF^C^ neutrophils, we hypothesize that the HIF-induced CXCL1 responses are not only required for chemotaxis of neutrophils, but also partially involved in inhibiting apoptosis during infection, perhaps consistent with the known angiogenic responses autocrine CXCL1 signaling has in epithelial cells [72], [73]. Recently, the implication for the requirement of neutrophil transmigration in the transcriptional imprinting of epithelial cells was demonstrated [74]. At the sites of transmigration, the depletion of oxygen by neutrophils in response to either a pathogen or tissue injury, resulted in the stabilization of HIF1α, which was required for mucosal protection and inflammatory resolution [74]. In addition, the release and sensing of VEGF in an autocrine and paracrine manner by monocytes, keratinocytes, and endothelial cells is required for wound healing responses to restore proper tissue homeostasis [75]. Tumors are notorious for exploiting this mechanism to aid in their growth and metastasis, as demonstrated by increased tumor growth with the paracrine signaling of CXCL1 in breast cancer [76]. The increased survival of the HIF^C^ neutrophils with exogenous CXCL1 supports the possibility that decreases in cytokine production in the HIF^C^ mice is due to decreased paracrine signaling responses between the deficient and less abundant myeloid cells and the epithelia/endothelia of the lung.

A direct translational outcome from our study that warrants further investigation is the observation that corticosteroids reduced nuclear localization and gene regulation of HIF1α. Given the phenotype of the HIF^C^ mice, it stands to reason that steroid mediated suppression of HIF1α could contribute to aspergillosis susceptibility. Our results demonstrate that steroid treatment does not inhibit overall HIF1α protein levels, but rather reduces the nuclear levels of HIF1α and p65 NFκB. The decrease in nuclear p65 NFκB and subsequent HIF1α mRNA abundance did not cause a decrease in HIF1α protein accumulation in the cytoplasm, indicating that there may be a separate corticosteroid induced NFκB-independent mechanism that hinders the HIF1α induced response to *A. fumigatus*. Nuclear import of HIF1α is known to rely upon interactions with the septin SEPT9_i1, a product of transcript SEPT9_v1 that encodes isoform1, and importin-α [77]. These interactions have been established in the context of tumor and cancer progression, but the interactions during steroid treatment are unknown to our knowledge, and a mechanism for the steroid blockage of HIF1α cytoplasm-nuclear localization is not yet understood. There may also be differential posttranslational regulation of HIF1α under corticosteroid conditions that is impacting the localization, interactions, and nuclear stability of HIF1α in myeloid cells. Interestingly, corticosteroid treated mice were demonstrated to have a two-fold reduction in CXCL1 production compared to immune competent mice following *A. fumigatus* challenge [78] further supporting a role for HIF1α in steroid induced IPA susceptibility.

Taken together, our data support the conclusion that myeloid derived HIF1α is required by effector cells of the innate immune system to prevent *A. fumigatus* pulmonary infection. As control of metabolism and production of energy in inflammatory, low oxygen infection sites is dependent upon HIF1α, it is in accord that innate inflammatory responses required for clearing and preventing infection are also tied to this important transcriptional regulator. It will be of translational importance to determine if this idea is reflected in the mechanisms underlying the susceptibility of certain patients to *A. fumigatus* infection. Consequently, it is a high priority to determine if this response can be targeted to reverse the inactive and suppressed effects of the innate cells during IPA in the context of corticosteroid mediated immune suppression. The success of HIF1α agonists in the context of bacterial skin infections is promising in this regard [23], [25], [79].

## Materials and Methods

### Fungal culture and growth conditions

*Aspergillus fumigatus* strain CBS144.89 (also known as CEA10) was used in all experiments except those involving the FLARE tdtomato strain generated from CEA17 (uracil auxotroph derived from CEA10). All strains were grown on glucose minimal medium with 1.5% agar at 37°C. Conidia were dislodged from plates with a cell scrapper, re-suspended in 0.01% Tween-20, and filtered through miracloth (EMD chemicals, CalBiochem).

### FLARE strain generation

*A. fumigatus* strain CEA17 was transformed with a construct consisting of an overlap PCR of the *gpdA* promoter driven-tdtomato from pSK536 (gift from Dr. Sven Krappmann) with *A. parasiticus pyrG*. The construct was ectopically inserted into the genome using the standard fungal protoplast transformation as previously described [80]. Transformants were initially screened by microscopy and flow cytometry for tdtomato expression. One of five transformants were selected for further use, based on bright fluorescence in all growth stages, comparable radial growth to parental strain, and comparable inflammatory TNF responses by BMDMs (Figure S3). Copy number was confirmed by Southern analysis with the digoxigenin labeling system (Roche Molecular Biochemicals, Mannheim, Germany) as previously described [81]. Generation of FLARE was done as previously described in [40] using Biotin conjugated AF633-streptavidin or Biotin conjugated BilliantViolet421-streptavidin (BV421) (BioLegend). For conidial kill assays, 2.5×10^5^ FLARE conidia were incubated in 0.2 ml RP10 with 0–10 M H_2_O_2_ for 30 min at 37°C, washed, and analyzed by flow cytometry for TdTomato and BV421 fluorescence. Duplicate samples were plated (at 1∶1,000 dilution) to determine the cfu.

### Murine models of invasive pulmonary aspergillosis

CD1 female mice, 6–8 weeks old were used in the corticosteroid (triamcinolone acetate, Kenalog) experiments. Mice were obtained from Charles River Laboratories (Raleigh, NC). For the corticosteroid model, mice were immunosuppressed with a single dose of Kenalog (Bristol-Myers Squibb Company, Princeton, NJ, USA) injected subcutaneously (s.c.) at 40 mg/kg 1 day prior to inoculation. For the immunocompetent experiments, mice 10–12 weeks old with targeted myeloid deletions of HIF1α created via crosses into a background of lysozyme M–driven cre (HIF^C^) expression and littermate controls (cre-/HIF1α floxed) were used as described in [22]. For infections, mice were lightly anesthetized and immobilized in an upright position using rubber bands attached to a Plexiglas stand for oropharyngeal aspiration. A blunt 20G needle attached to a 1 ml syringe was advanced into the trachea to deliver the indicated number of conidia (3–7×10^7^) in a volume of 0.05 ml PBS or PBS with 0.025% Tween-20.

### Western analysis

Nuclear and cytoplasmic proteins were isolated from lyophilized lung tissue, BMDMs, or J774.1 macrophages at indicated times. Cells were centrifuged at 1500 rpm for 5 min at 4°C. Supernatant was removed, and the pellet was washed with 5 packed cell volumes (PCV) of buffer A [10 mM Tris-HCl (pH 7.5), 1.5 mM MgCl_2_, 10 mM KCl supplemented with 1M dithiothreitol, 0.2 M PMSF, 1 mg/ml leupeptin, 1 mg/ml aprotinin, 1 mg/ml pepstatin, and 0.5 M Na_3_VO_4_], resuspended in 4 PCV of buffer A and incubated on ice for 10 min. The cell suspension and lung tissue were homogenized, and nuclei were pelleted by centrifugation at 10,000 g for 10 min at 4°C and the supernatant was collected as the cytoplasmic fraction. The cell pellet was resuspended in 3 PCV of buffer C [20 mM Tris-HCl (pH 7.5), 0.42 M KCl, 1.5 mM MgCl_2_, and 20% glycerol] and rotated for 30 min at 4°C. The suspension was centrifuged at 20,000 g for 10 min at 4°C. The protein concentration was determined using the Bradford method (Bio-Rad, Hercules, CA, USA). Nuclear and cytoplasmic samples were suspended in 6× SDS sample buffer, boiled for 10 min, and loaded onto a 10% mini-protein precast gels (Bio-Rad) for SDS-PAGE. After gel electrophoresis, protein was transferred to a PVDF membrane using the trans-blot turbo transfer system (Bio-Rad). HIF1α and NFκB (p65) were detected using polyclonal rabbit anti-mouse antibodies NB100-449 (1∶3000) and C-20:sc-372 (1∶1200), respectively and an anti-rabbit HRP-conjugated secondary antibody raised in goat (millipore) at a 1∶5000 dilution. Chemiluminescence was measured following incubation of blots with Clarity Western ECL substrate (Bio-Rad) using a FluorChem FC2 imager (Alpha Innotech). For loading controls, anti-tubulin (Sigma, T5192) (human) was utilized.

### Real-time RT-PCR

Tissue or BMDMs were re-suspended in Trizol reagent and chloroform to extract RNA. RNA was DNase treated with DNA-free kit (Ambion) and reverse transcribed with QuantiTect reverse transcription kit (Qiagen, USA). Primers for all murine genes of interest were designed with PrimerQuest (IDT) and manufactured by IDT, USA. Sequences are: *hif1α* fwd: ATGAGATGAAGGCACAGA, rev: CACGTTATCAGAAATGTAAACC, *cxcl1 (kc)* fwd: TGCACCCAAACCGAAGTCAT, rev: TTGTCAGAAGCCAGCGTTCAC, *cxcr2* fwd: TGGCCTAGTCAGTCATCA, rev: CAATCCACCTACTCCCATTC, *tlr4* fwd: GTGTGTGTGTGTGTGTTG, rev: AGCTGCTCTGTACACTATTT, *dectin1 (clec7a)* fwd: CCTAGTGTGATCTGTCTTGT, rev: TTTCTGCCCACATATTGATTAG, *hprt* fwd: GGAGTCCTGTTGATGTTGCCAGTA, rev: GGGACGCAGCAACTGACATTTCTA, *rpl13a* fwd: CTCTGGAGGAGAAACGGAAGGAAA, rev: GGTCTTGAGGACCTCTGTGAACTT. All reactions were performed on BioRad MyIQ real-time PCR detection system with IQ SYBR green supermix (Bio-Rad, Hercules, CA). The ΔΔC_t_ method was used to assess changes in mRNA abundance, using either *hprt* or *rpl13a* as the housekeeping gene. Results presented are the mean and standard deviation from 3 biological and 3 technical replicates.

### Analysis of *A. fumigatus* challenged mice

*A. fumigatus* challenged mice were euthanized at indicated times. For histological studies, the lungs were inflated with 10% buffered formalin, fixed, and embedded in paraffin to generate 4 µm sections stained with hematoxylin and eosin (H&E) or Gomori's Methenamine Silver (GMS) stain for microscopy by the Dartmouth Immunology COBRE core facility or at Montana State University. Contiguous tissue sections were imaged using a Zeiss Axioscope 2-plus microscope and imaging system (Zeiss, Jena, Germany) and a Leica upright DMRXA2 with Leica application suite software and DC500 camera (Leica Microsystems, Buffalo Grove, IL, US). Pathological examination was conducted for apoptosis, necrosis, and vasculature observation. Image analysis was performed using ImageJ software (v.1.46i).

For immunohistological studies, the left lung of each mouse was filled with OCT (frozen tissue matrix) and after embedding in OCT immediately frozen in liquid nitrogen. The lungs were stained as previously described in [21], [82] with a rabbit polyclonal antibody to Aspergillus (Abcam Inc., Cambridge, MA, USA) and detected with AlexaFluor488-conjugated goat Anti-rabbit (Invitrogen, Carlsbad, CA, USA) diluted 1∶400. After another washing step, prolong Gold antifade reagent with DAPI (Invitrogen, Carlsbad, CA, USA) was added to each section. Microscopic examinations were performed on a Zeiss Axioscope 2-plus microscope and imaging system (Zeiss, Jena, Germany). For each time point, a total of 2 to 4 mice were examined and experiments were repeated in triplicate.

To assess fungal burden in lungs, mice were sacrificed at 24, 36, or 48 hrs post inoculation, and lungs were harvested and immediately frozen in liquid nitrogen. Samples were freeze-dried, homogenized with glass beads on a Mini- Beadbeater (BioSpec Products, Inc., Bartlesville, OK, USA), and DNA extracted with the E.N.Z.A. fungal DNA kit (Omega Bio- Tek, Norcross, GA, USA) or phenol chloroform extraction. Quantitative PCR was performed as described previously [39].

Cellularity was analyzed on cells from the BALF at specific time points of 4, 8, or 12 hrs. Cells isolated from BALFs were enumerated and stained with the following Abs: anti-CD11b (clone M1/70), anti-CD11c (clone N418), and anti-Ly6G (clone 1A8) in staining buffer (PBS supplemented with 2% FBS). Neutrophils were identified as CD11b^+^CD11c^−^Ly6G^hi^, macrophages as CD11c^+^CD11b^−^Ly6G^−^, and inflammatory monocyte-like cells as CD11b^+^CD11c^−^Ly6G^lo^ (negative for NK1.1 staining, data not shown). A fourth population of cells staining with CD11c^+^CD11b^+^Ly6G^+^ were found, but further determination was not pursued. Flow cytometry data were collected on a MACSQuant 10 (Miltenyi Biotec) and analyzed with FlowJo, v.9.4.3 (TreeStar).

To assess the requirement of CXCL1 for *in vivo* infection, HIF^C^ mice received 50 µL of 50 ng recombinant CXCL1 (BioLegend) 4 hrs following inoculation of 7×10^7^ conidia and were compared to HIF^C^ mice and WT infected mock mice receiving PBS. Separate experiments analyzing survival, cell recruitment by flow cytometry, and fungal burden were conducted.

Single-cell lung suspensions were prepared for flow cytometric analysis and classified as described in Hohl et al. (2009). Tissue processing did not result in leukocyte uptake of exogenously added FLARE conidia. Lung digest and, if applicable, BALF cells were enumerated and stained with the following Abs: anti-Ly6C (clone AL-21) anti-Ly6G (clone 1A8), anti-CD11b (clone M1/70), anti-CD45.2 (clone 104), and anti-Ly6B.2 (clone 7/4). PE- and APC-labeled Abs were omitted in FLARE experiments. Neutrophils were identified as CD45^+^CD11b^+^Ly6C^lo^Ly6G^+^Ly6B.2^+^ cells. Flow cytometry data were collected on a BD LSR II flow cytometer or MACSQuant 10 (Miltenyi Biotec) and analyzed with FlowJo, v.9.4.3 (TreeStar).

### LDH and Albumin assays

Lactate dehydrogenase (LDH) using the CytoTox96 non-radioactive cytotoxicity assay kit (Promega, Cat. No. 573702) and albumin assay (Albumin (BCG) Reagent Set, Eagle Diagnostics, Cedar Hill, TX, USA) were conducted on BALFs from WT and HIF^C^ mice inoculated with 7×10^7^ conidia or PBS according to manufacturers instructions with slight variation. Briefly, 100 µL of the BALF was added to equal volumes of the respective agents and incubated for either 30 min (LDH) or 5 min (Albumin) and read at 490 nm and 630 nm, respectively. Albumin levels were determined using a standard curve and LDH values for each time point are relative to the WT PBS BALF sample.

### Bone-marrow derived cells

Bone marrow (BM) cells were eluted from tibias and femurs of 8–12 week old Littermate or HIF^C^ mice, lysed of red blood cells, and cultured for macrophages in RP20 (RPMI, 20% FCS, 5 mM HEPES buffer, 1.1 mM L-glutamine, 0.5 U/ml penicillin, and 50 mg/ml streptomycin) supplemented with 30% (v/v) L929 cell supernatant (source of M-CSF) or neutrophils in murine neutrophil buffer (HBSS containing 0.1% FBS and 1% glucose). BM cells for macrophages were plated in a volume of 20 ml at a density of 2.5×10^6^ cells/ml in 10 ml petri dishes. The medium was exchanged on day 3. Adherent BM-derived macrophages (BMDMs) were harvested on day 6. BM- derived cells for neutrophils (BMDNs) were suspended in 3 ml 45% percoll and isolated from a 30 min 1600× g percoll gradient (top to bottom: 3 ml 45% percoll containing BM cells, 2 ml 50%, 2 ml 55%, 2 ml 62%, and 3 ml 81%) in a Sorvall Legend Mach 1.6R benchtop centrifuge, with a BIOshield 600 rotor-75002005 (Thermo Scientific). BMDNs were collected from the 62/81% border and washed with HBSS before live cell counting (95% purity, determined by cytospin). For the cytokine mRNA abundance quantifying experiments, BMDMs were incubated with *A. fumigatus* conidia (strain CBS144.89) in a 10∶1 (effector∶target) ratio 8 hrs. Following the incubation, cells were directly re-suspended in Trizol reagent and chloroform to extract RNA for qRT-PCR.

### CFU and XTT assays

BMDMs were incubated with *A. fumigatus* conidia in a 9∶1 (effector∶target) ratio 3 hrs (for CFU) and 16 hrs (for XTT). Following the 3 hr incubation, non-phagocytosed conidia were washed off the cells, serially diluted onto GMM plates in duplicate, and CFU was determined. Following the 16 hr incubation, BMDMs were cold water lysed and the percent damage was quantitated by measuring the OD at 450 nm following a 1 hr incubation with XTT as previously described [82].

### Cytokine analysis

Collected BALFs were assayed by ELISA and luminex. Commercially available ELISA kits for CXCL1 (Assay Biotech, OK-0189), CXCL2 (R&D systems, DY452), and CXCL5 (R&D Systems, DY443) were used according to the manufactures' instructions. The limit of detection was 15 pg/ml. Luminex analysis was carried out using Bio-Plex Pro Mouse Cytokine immunoassay on a Bio-Plex Array Reader (Bio-Rad Laboratories Inc., Hercules, CA) according to the manufactures' instructions. Bio-Plex Manager software with five-parametric-curve fitting was used for data analysis and procedure was carried out by the Dartmouth Immunology COBRE core. For BMDM cytokine analysis of TdTomato strain, cells were washed and plated in 0.2 ml TC medium at a density of 5×10^5^ cells/ml in 96 well plates and co-incubated in a 9∶1 ratio with conidia for 8 hrs. Supernatants were collected for ELISA. A commercially available ELISA kit for TNF (eBioscience, San Diego, California, US) was used according to the manufactures' instructions. The limit of detection was 15 pg/ml for TNF.

### Migration assay

BMDN migration was examined using Costar Transwell plates (6.5 mm diameter insert, 3.0 µm pore size, polycarbonate membrane, Corning Inc., Corning, NY). To determine if migration was defective, 10% FBS was added to the bottom chamber of these plates (media without FBS was used as a migration control). Isolated BMDNs were counted using trypan blue (Sigma), then placed in serum free medium (SFM). Cells were resuspended at 1×10^6^/ml SFM and 250 µl were allowed to migrate for 3 hr at 37°C at 5% CO_2_. Following migration, the medium in the top chamber was aspirated and the membrane gently wiped with a cotton swab to remove the cells that did not migrate. The membranes were first rinsed with PBS, the cells were then fixed with 2% formaldehyde in PBS, permeabilized with 0.01% Triton X-100 (Sigma) in PBS and finally stained with crystal violet (Sigma). Cells that migrated across the membrane were counted. Ten random fields at 40× were counted for each condition using light microscopy. Each experiment was repeated three times. Results are expressed as mean cell migration normalized to media control ± SEM. To determine the role for cytokine signaling in migration defects, a BALF-switch experiment using BALFs from infected HIF^C^ and littermate mice in the bottom chamber was conducted. Briefly, 250 µl BMDNs at 1×10^6^/ml SFM were added to the top chamber of a transwell plate with FN coated membranes with 300 µl of BALF in the bottom chamber and were allowed to migrate for 3 hr at 37°C and 5% CO2. For add-back experiments, recombinant CXCL1 (Biolegend) was added back to the BALFs from infected HIF^C^ mice to the concentration determined by ELISA in the infected littermate BALFs (600 pg).

### Neutrophil apoptosis

Neutrophil apoptosis was measured using FACs analysis by staining BMDN's incubated at 37°, 5% CO_2_ in RP20 medium with annexin V-Pacific Blue (PB) (BioLegend, #640917) and PI (millipore). Staining was performed by following the manufacturer's instructions, with minor changes. Briefly, after isolation or incubation for the specified time points, neutrophils were washed twice with ice-cold PBS with 2% FBS and then resuspended in Annexin-V binding buffer (0.01 M HEPES, pH 7.4; 140 mM NaCl; 2.5 mM CaCl_2_). Annexin V-PB and PI were added into the culture tube and incubated for 15 min prior to direct analysis with flow cytometry. Viable neutrophils were defined as negative for annexin V-PB and PI staining; apoptotic neutrophils were defined as positive for annexin V-PB staining but negative for PI staining. Cells positive for both annexin V-PB and PI staining were considered necrotic cells. Cell survival/apoptosis was expressed as a percentage of neutrophils relative to the total number of counted neutrophils. *In vivo* neutrophils were quantified through analysis of BALF cytospins as previously described [45], [46], [47]. Briefly, apoptotic neutrophils were visualized by counting the number of neutrophils with pyknotic nuclei and karyorrhexis out of the total number of neutrophils per frame. Ruffled neutrophils were also quantified and depicted neutrophil activation. Ten random frames at 8 hrs post challenge were analyzed from four HIF^C^ and four WT mice (analyzed two separate experiments).

### Ethics statement

This study was carried out in strict accordance with the recommendations in the Guide for the Care and Use of Laboratory Animals of the National Institutes of Health. The animal experimental protocol was approved by the Institutional Animal Care and Use Committee (IACUC) at Dartmouth College (protocol number cram.ra.1).

## Supporting Information

Figure S1 **Analysis of HIF1α protein abundance in J774.1 macrophages and in macrophages from mice deficient in myeloid HIF1α.** A) Nuclear protein abundance of HIF1α from J774.1 macrophages incubated with *A. fumigatus* conidia, germlings, hyphae, or nothing for 6 hrs in normoxic conditions. B) Protein abundance of HIF and NFκB p65 subunit in the cytoplasmic and nuclear extracts from WT and HIF^C^ BMDMs incubated with and without conidia (10∶1 ratio) for 8 hrs. Bar graph depicting quantitation of the band densities for HIF and p65 following normalization to γ-tubulin (γ-tub).
(TIFF)

Figure S2 **Histology and immunohistochemistry of HIF^C^ and WT mice (to go with** **Fig. 2** **).** Immune competent littermate (WT) and HIF^C^ mice received 7×10^7^ conidia i.t. (A) Representative histology of H&E or GMS stained lung sections from HIF^C^ and WT mice at 8 hr post challenge. Left image (5×), right image (20×). (B) Representative immunohistochemistry of WT and HIF^C^ mice 5 µm frozen lung sections stained with anti-aspergillus (green) and DAPI (blue) at 24 and 48 hr post challenge. All images are 20×.
(TIFF)

Figure S3 **FACs analysis, TNF stimulation, and viability test of CEA10 FLARE strain.** FACs analysis demonstrating the different components and scatter profiles of the FLARE construction: Conidia from CEA10 WT strain (A), CEA10 strain with biotin conjugated BV421-SA (B), TdTomato CEA10 strain (C), and TdTomato strain with biotin conjugated BV421-SA (FLARE) (D). FACs plots are BrilliantViolet (y-axis) vs. TdTomato (x-axis). (E) ELISA for TNF protein on supernatants from WT BMDM's incubated with TdTomato and CEA10 conidia for 8 hr. (F) The graph shows TdTomato (squares) and BV421 (triangles) fluorescence and cfu (circles) from FLARE conidia exposed to the indicated H_2_O_2_ concentration demonstrating the TdTomato instability and BV421 stability when encountering oxidative stress. Mean fluorescence intensity is indicated relative to FLARE conidia not treated with H_2_O_2_.
(TIF)

Figure S4 **Flow cytometry dot plot to go with** **Fig. 7** **. **Representative FACs analysis of cell recruitment (same staining and gating strategy as in Figure 4) at 8 hrs post challenge using CD11b, Ly6G, and CD11c fluorescent antibodies for staining of cells in the lung BALF of WT, HIF^C^, and HIF^C^ mice infected with 7×10^7^ conidia and treated with PBS or 50 ng rCXCL1 4 hr post conidial challenge. Main plots are CD11b v. CD11c and expanded cell population gate I plot is CD11b v. Ly6G.
(TIF)

Figure S5 **Putative HIF1α binding sites in CXCL1 promoter and PRR expression profiles on neutrophils.** (A) Nucleotide sequence from the mouse CXCL1/KC gene containing the 5″flanking region with ∼480 nucelotides from the indicated transcriptional start site [70], [83]. Known NFκB and putative HIF1α binding motifs are indicated in the promoter region. (B) WT and HIF^C^ BMDNs were incubated with *A. fumigatus* conidia in a 10∶1 ratio for 3.5 hrs. mRNA abundance of *cxcr2, tlr4, and dectin1* was determined using quantitative RT-PCR, normalized to *rpl13a*, and relative to the WT sample (2 biological and 3 technical replicates).
(TIFF)
