# Supplementary figures and images for: Myeloid Derived Hypoxia Inducible Factor 1-alpha Is Required for Protection against Pulmonary Aspergillus fumigatus Infection
Source: PLoS Pathog. 2014 Sep 25;10(9):e1004378. doi: 10.1371/journal.ppat.1004378 (PMC4177996; doi:10.1371/journal.ppat.1004378)

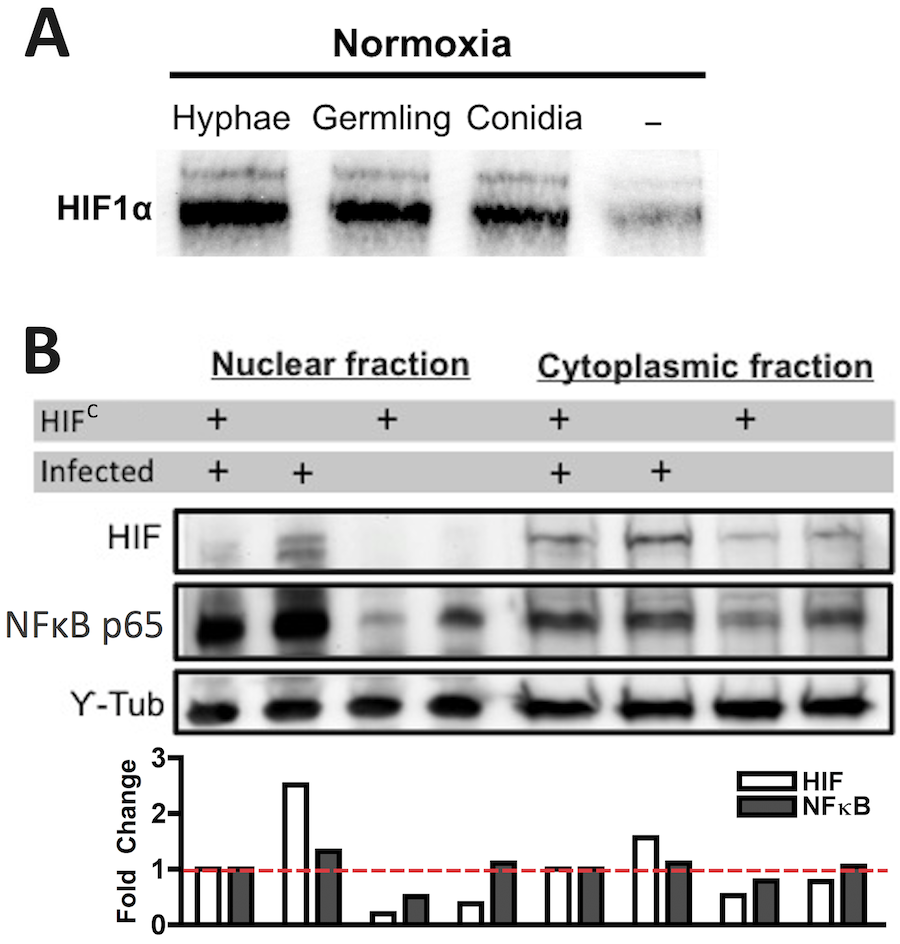

Supplement: Figure S1 — Analysis of HIF1α protein abundance in J774.1 macrophages and in macrophages from mice deficient in myeloid HIF1α. A) Nuclear protein abundance of HIF1α from J774.1 macrophages incubated with A. fumigatus conidia, germlings, hyphae, or nothing for 6 hrs in normoxic conditions. B) Protein abundance of HIF and NFκB p65 subunit in the cytoplasmic and nuclear extracts from WT and HIFC BMDMs incubated with and without conidia (10∶1 ratio) for 8 hrs. Bar graph depicting quantitation of the band densities for HIF and p65 following normalization to γ-tubulin (γ-tub). (TIFF) [file ppat.1004378.s001.tiff]

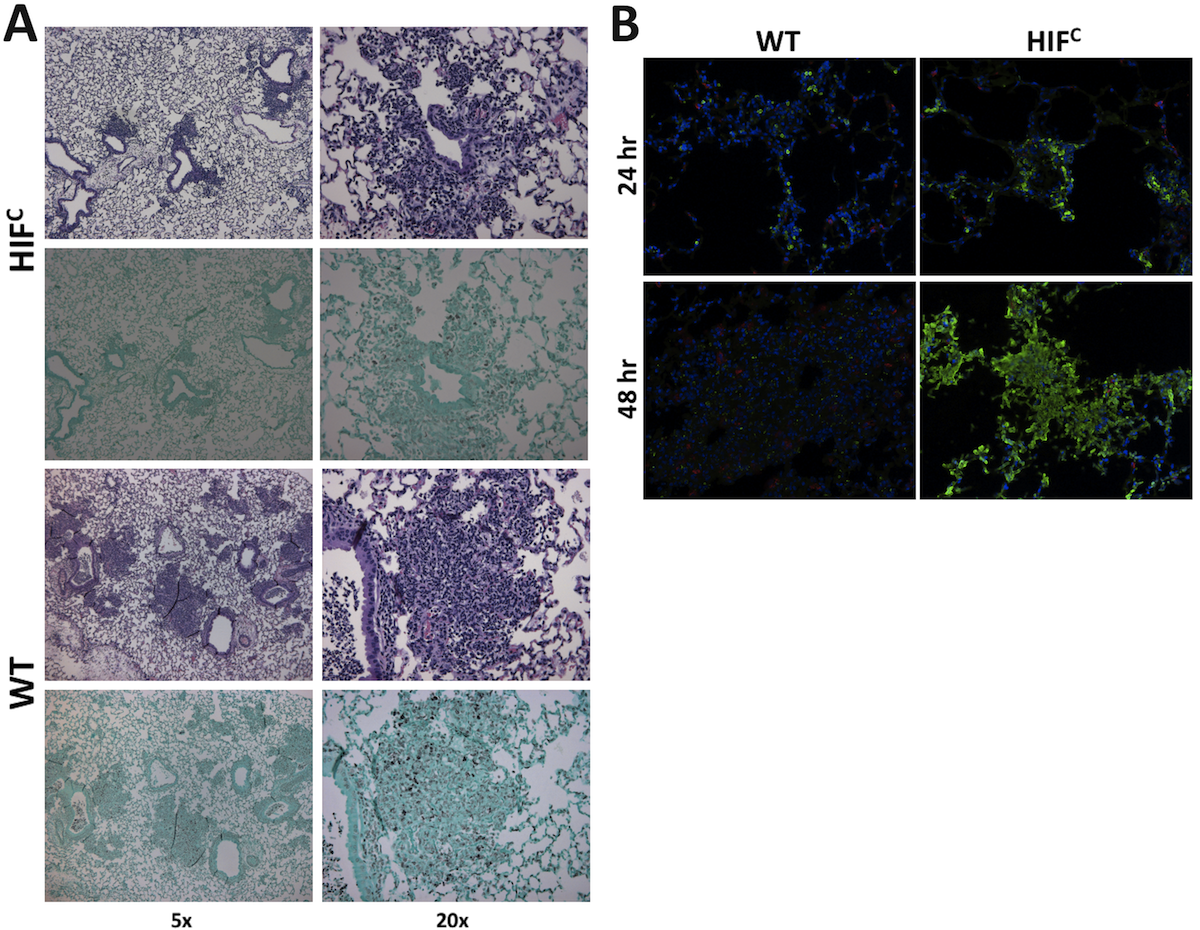

Supplement: Figure S2 — Histology and immunohistochemistry of HIFC and WT mice (to go with Fig. 2 ). Immune competent littermate (WT) and HIFC mice received 7×10^7^ conidia i.t. (A) Representative histology of H&E or GMS stained lung sections from HIFC and WT mice at 8 hr post challenge. Left image (5×), right image (20×). (B) Representative immunohistochemistry of WT and HIFC mice 5 µm frozen lung sections stained with anti-aspergillus (green) and DAPI (blue) at 24 and 48 hr post challenge. All images are 20×. (TIFF) [file ppat.1004378.s002.tiff]

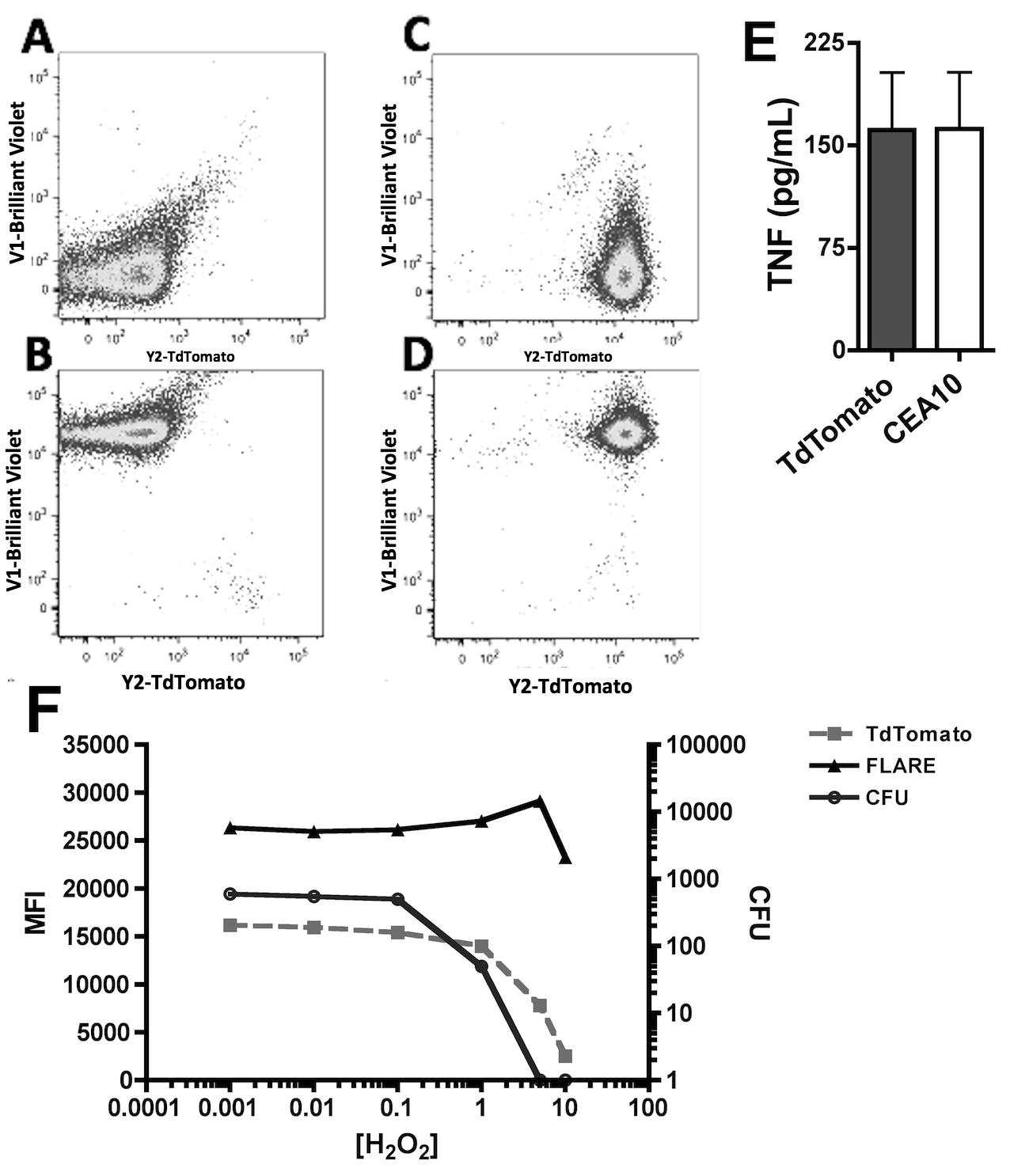

Supplement: Figure S3 — FACs analysis, TNF stimulation, and viability test of CEA10 FLARE strain. FACs analysis demonstrating the different components and scatter profiles of the FLARE construction: Conidia from CEA10 WT strain (A), CEA10 strain with biotin conjugated BV421-SA (B), TdTomato CEA10 strain (C), and TdTomato strain with biotin conjugated BV421-SA (FLARE) (D). FACs plots are BrilliantViolet (y-axis) vs. TdTomato (x-axis). (E) ELISA for TNF protein on supernatants from WT BMDM's incubated with TdTomato and CEA10 conidia for 8 hr. (F) The graph shows TdTomato (squares) and BV421 (triangles) fluorescence and cfu (circles) from FLARE conidia exposed to the indicated H2O2 concentration demonstrating the TdTomato instability and BV421 stability when encountering oxidative stress. Mean fluorescence intensity is indicated relative to FLARE conidia not treated with H2O2. (TIF) [file ppat.1004378.s003.tif]

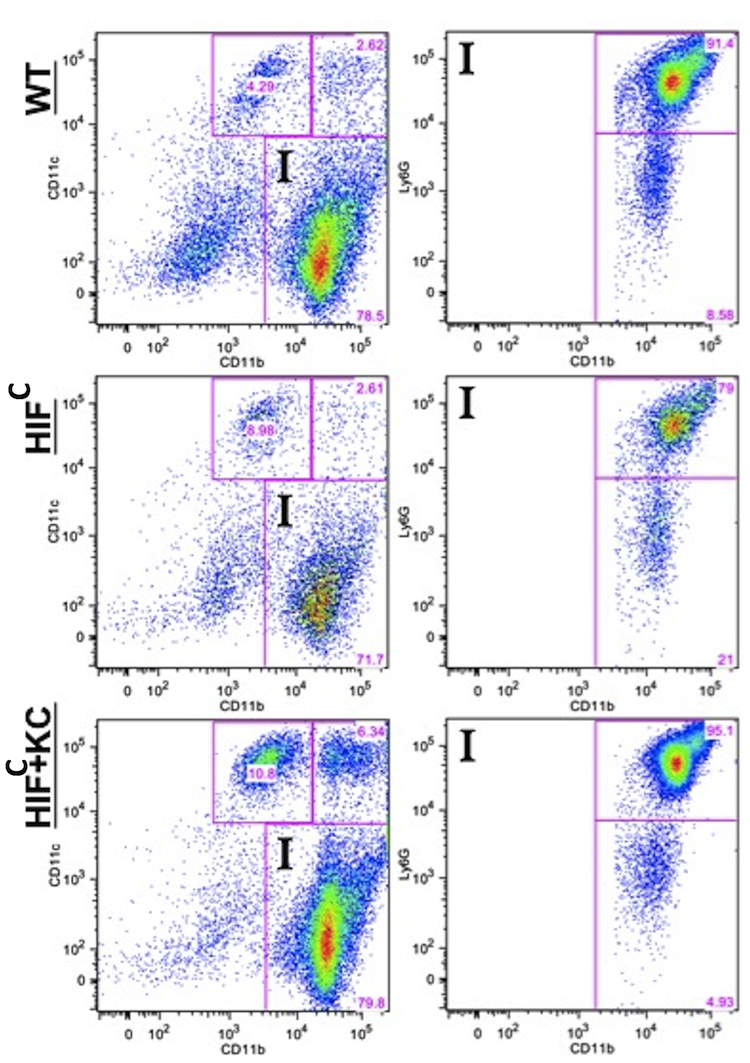

Supplement: Figure S4 — Flow cytometry dot plot to go with Fig. 7 . Representative FACs analysis of cell recruitment (same staining and gating strategy as in Figure 4) at 8 hrs post challenge using CD11b, Ly6G, and CD11c fluorescent antibodies for staining of cells in the lung BALF of WT, HIFC, and HIFC mice infected with 7×10^7^ conidia and treated with PBS or 50 ng rCXCL1 4 hr post conidial challenge. Main plots are CD11b v. CD11c and expanded cell population gate I plot is CD11b v. Ly6G. (TIF) [file ppat.1004378.s004.tif]

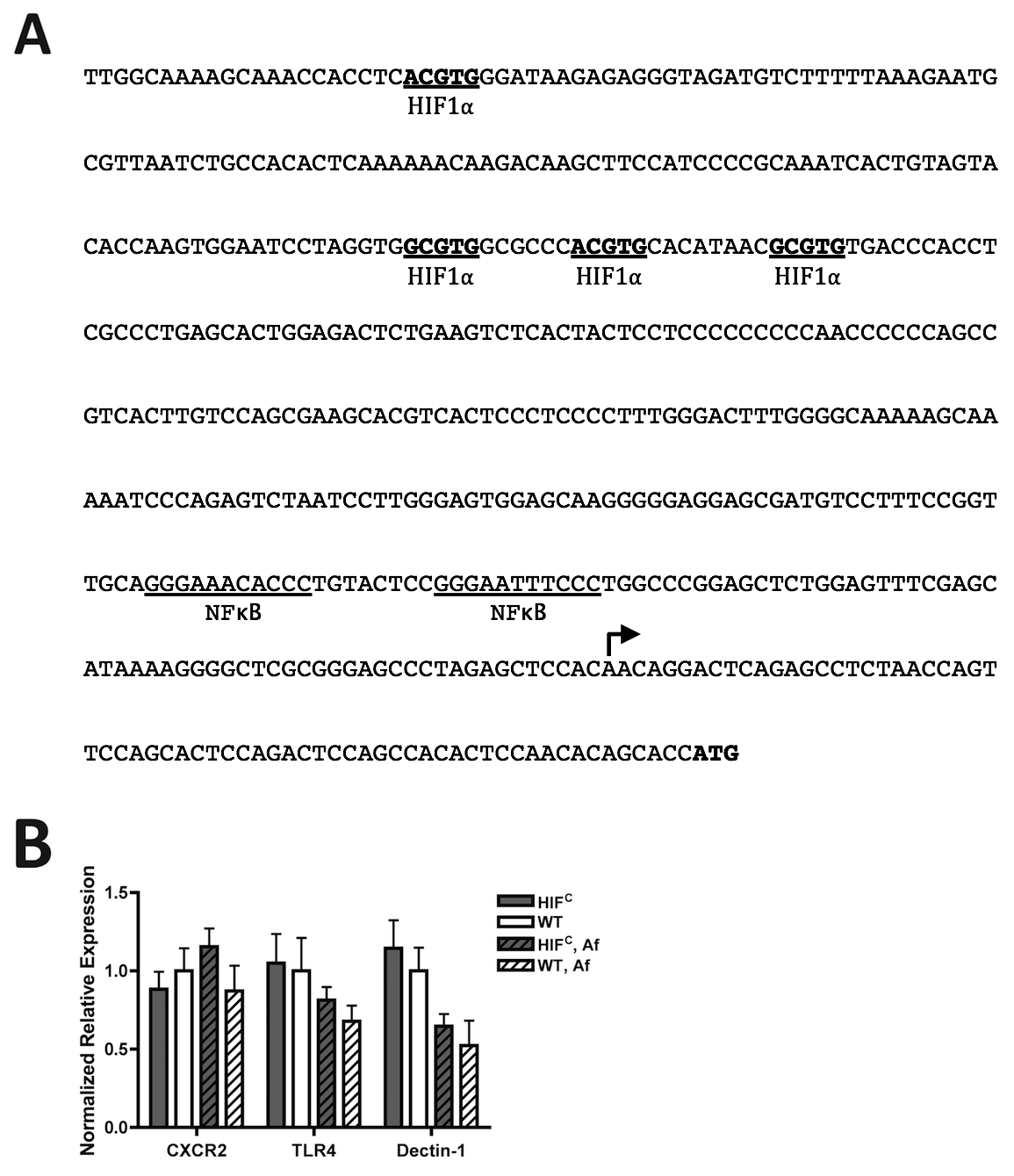

Supplement: Figure S5 — Putative HIF1α binding sites in CXCL1 promoter and PRR expression profiles on neutrophils. (A) Nucleotide sequence from the mouse CXCL1/KC gene containing the 5″flanking region with ∼480 nucelotides from the indicated transcriptional start site [70], [83]. Known NFκB and putative HIF1α binding motifs are indicated in the promoter region. (B) WT and HIFC BMDNs were incubated with A. fumigatus conidia in a 10∶1 ratio for 3.5 hrs. mRNA abundance of cxcr2, tlr4, and dectin1 was determined using quantitative RT-PCR, normalized to rpl13a, and relative to the WT sample (2 biological and 3 technical replicates). (TIFF) [file ppat.1004378.s005.tiff]
